# King's College Hospital Removal Fund

**Published:** 1903-11-14

**Authors:** W. F. D. Smith, E. S. Roffen, Charles Awdry, Arthur C. Headlam, David Ferrier, W. Watson Cheyne

**Affiliations:** Chairman of the Joint Committee Removal of King's College Hospital.; Lieut.-General, Chairman of the Hospital Committee.; Vice-President and Consulting Surgeon of the Hospital.; Treasurer.; Principal of King's College.; Senior Physician of the Hospital.; Senior Surgeon of the Hospital.


### EDITOR'S LETTER-BOX.

[Our Correspondents are reminded that prolixity is a great bar to publication, and that brevity of style and conciseness of statement greatly facilitate early insertion.]

### KING'S COLLEGE HOSPITAL REMOVAL FUND.

Sir,—As already reported in your columns, at a meeting of the Governors held on October 12th, 1903, it was finally decided to remove King's College Hospital from its present position to a site in South London.

The main object of the removal is more fully to carry out the idea of the Charity by transferring it from a district largely denuded of its poorer inhabitants to one in which there is a dense population urgently in need of hospital accommodation. By the removal this urgent need, which has long existed in South London, will be met, without the addition of another large general hospital to those at present existing, most of which already have a constant struggle in meeting their necessary expenditure.

The Governors when sanctioning the present course authorised the joint committee appointed by the Council of King's College and the Committee of Management of the Hospital to act for them in all matters appertaining to the removal. In accepting this authority the committee are fully conscious of the great responsibility they have taken upon themselves, and they are determined to jealously guard the traditions of the Charity, and the many benefactions made to it.

A sum of not less than £300,000 will be required to build and equip the hospital, so as to enable it to meet the needs of the three-quarters of a million of population who will be benefited by the removal. The committee now appeal to the public for help towards raising this required sum, and they feel confident that they will not appeal in vain. They are allowed to add that this appeal is issued with the approval of his Royal Highness the Duke of Cambridge, President of the hospital. It also has the cordial support and hearty approval of his Grace the Archbishop of Canterbury, one of the Vice-Presidents of the hospital and visitor of the college. A suitable site of 12 acres in South London has been presented to the hospital.

It remains but to say that donations to the Removal Fund will be thankfully received by the treasurer, Charles Awdry, Esq., 2 Hyde Park Street, W.; by Lloyd's Bank, Limited, Law Courts branch, 222 Strand, bankers to the hospital; and at King's College, Strand, by A. MacLeod Forest, Esq., the secretary King's College Hospital Removal Fund.

W. F. D. Smith, Chairman of the Joint Committee Removal of King's College Hospital.
Methuen, Lieut.-General, Chairman of the Hospital Committee.
E. S. Roffen.
Lister, Vice-President and Consulting Surgeon of the Hospital.
Charles Awdry, Treasurer.
Arthur C. Headlam, Principal of King's College.
David Ferrier, Senior Physician of the Hospital.
W. Watson Cheyne, Senior Surgeon of the Hospital.

